# Identification of novel biomarkers in ischemic stroke: a genome-wide integrated analysis

**DOI:** 10.1186/s12881-020-00994-3

**Published:** 2020-03-30

**Authors:** Qizhi Xie, Xiaoyun Zhang, Sijia Peng, Jingjing Sun, Xiao Chen, Yuanfei Deng, Li Yi

**Affiliations:** 1 Department of Neurology, Peking University Shenzhen Hospital, Shenzhen, China; 2 Department of Clinical Medicine, Shantou University Medical College, Shantou, China; 3 National Clinical Research Center for Geriatric Diseases Shenzhen Center, Peking University Shenzhen Hospital, Shenzhen, China

**Keywords:** Ischemic stroke, Bioinformatics, Biomarkers, Genes, miRNA, Atypical infection

## Abstract

**Background:**

Ischemic Stroke (IS) is the most common neurological emergency disease and has become the second most frequent cause of death after coronary artery disease in 2015. Owing to its high fatality rate and narrow therapeutic time window, early identification and prevention of potential stroke is becoming increasingly important.

**Methods:**

We used meta-analysis and bioinformatics mining to explore disease-related pathways and regulatory networks after combining messengerRNA (mRNA) and miRNA expression analyses. The purpose of our study was to screen for candidate target genes and microRNA(miRNA) for early diagnosis of potential stroke.

**Results:**

Five datasets were collected from the Gene Expression Omnibus (GEO) database by systematical retrieval, which contained three mRNA datasets (102 peripheral blood samples in total) and two miRNA dataset (59 peripheral blood samples). Approximately 221 different expression(DE) mRNAs (155 upregulated and 66 downregulated mRNAs) and 185 DE miRNAs were obtained using the metaDE package and GEO2R tools. Further functional enrichments of DE-mRNA, DE-miRNA and protein-protein interaction (PPI) were performed and visualized using Cytoscape.

**Conclusion:**

Our study identified six core mRNAs and two regulated miRNAs in the pathogenesis of stroke, and we elaborated the intrinsic role of systemic lupus erythematosus (SLE) and atypical infections in stroke, which may aid in the development of precision medicine for treating ischemic stroke. However, the role of these novel biomarkers and the underlying molecular mechanisms in IS require further fundamental experiments and further clinical evidence.

## Background

Stroke is the most common neurological emergency disease and has become the second leading cause of death after coronary artery disease in 2015, leading to 6.3 million deaths [1]. In addition, stroke is also a leading cause of long-term disability. The pathophysiological hallmarks of ischemic stroke involve part of the brain losing blood supply, which initiates the ischemic cascade. Brain tissue ceases to function if oxygen deprivation persists for 60 to 90 s, and will suffer irreversible death of brain cells occurs after approximately 3 h. The primary risk factor for stroke is hypertension; other risk factors include smoking, obesity, hyperlipidemia, diabetes, previous transient ischaemic attack and atrial fibrillation [2]. Stroke is characterized by neurological defect signs and symptoms, including hemiplegia, hemianesthesia, difficulty in speaking and understanding or loss of vision on one side. Even after intensive therapy, certain symptoms can be permanent, affecting 75% of stroke survivors and rendering them unable to manage their daily lives [3].

Stroke was originally deemed to be a sporadic disease. However, several epidemiology studies have shown that the morbidity of stroke or transient ischaemic attack was 12.3% among first degree relatives of stroke patients (vs 7.5% in the control group) [4], and the prevalence of stroke in offspring was shown to be three times higher if a parent had a stroke before 65 years of age [5]. Currently, it is widely believed that stroke is a complex multifactorial disease that is caused by interactions among blood vessels and environmental and genetic factors. Pathogenic mutations, such as Neurogenic locus notch homolog protein 3(*NOTCH3) gene* and HtrA Serine Peptidase 1(*HTRA1) gene*, have been reported in certain types of monogenetic stroke syndromes, such as cerebral autosomal dominant arteriopathy with subcortical infarcts and leukoencephalopathy(CADASIL) and cerebral autosomal recessive arteriopathy with subcortical infarcts and leukoencephalopathy(CARASIL). In addition, certain molecular genetic variations have been shown to be closely related to ischemic stroke, such as Paired-like homeodomain transcription factor 2 (*PITX2*), Histone deacetylase 9 (*HDAC9*), and Zinc finger homeobox protein 3 (*ZFHX3*) [5]. However, most of these gene mutations may exist as susceptibility genes, cooperating with other risk factors to cause the disease.

In addition to a single gene mutation, epigenetic mechanisms, such as DNA methylation, histone modifications and regulation by miRNAs, can also influence gene expression, which makes it difficult to analyze this disease, particularly sporadic stroke. Moreover, miRNA has been reported as a vital regulatory mechanism for the recovery of stroke [6] and has also been associated with the death of neurons and the repair of damaged tissue in the case of cerebral infarction [7]. Since the relationship between a given mi-RNA and its target genes is one-to-many rather than one-to-one, the mutual regulatory network between them may offer us a unique perspective to understand the disease and may provide potential therapeutic targets.

The massively parallel microarray technique can be applied to identify variant gene expression and pathways. This technique is used to investigate the relationship between gene expression and phenotypic differences and to gain deeper insights into the pathogenesis of complex diseases [8]. Bioinformation mining allows for the categorization and detection of large-scale genetic data according to phenotypic characteristics, potentially leading to novel hypotheses about the underlying mechanisms [9]. However, genome-wide expression data have limitations, such as small sample sizes, including poor repeatability and contradictory results. To take advantage of the big data era and reduce the limitations due to a small sample size, data from multiple datasets and platforms were collected in our integrated analysis.

The purpose of our study was to screen for candidate target genes and miRNAs in stroke using meta-analysis and bioinformatic mining and to explore disease-related pathways and regulatory networks after combining the mRNA and miRNA expression analyses.

Our study identified six core mRNAs and two regulated miRNAs in the pathogenesis of stroke, and we elaborated the intrinsic role of SLE and atypical infections in stroke.

## Methods

### Data collection and pre-processing

Expression profile data associated with stroke were obtained from Gene Expression Omnibus (GEO), which is a public functional genomic data repository. Ischemic stroke-related datasets were retrieved using the keyword “stroke” of *Homo sapiens* (organisms). Using the cutoff date August 15, 2018, 1037 datasets were retrieved. The inclusion criteria were as follows: (1) original experimental studies; (2) peripheral blood sample data provided; (3) mRNA expression profile provided; (4) access to the raw data (CEL files); and (5) the required diagnostic criteria for ischemic stroke are fulfilled. The exclusion criteria were as follows: (1) non-ischemic stroke sample; (2) repeated uploading of datasets; and (3) retrospective analysis. All of the included analyses were verified by the ethics committee. Pre-processing programs (including background adjustment, normalization, summarization, gene chip probe annotation) were executed using R language. CEL files were loaded using library (affy) to read the signal diagrams. We use the RMA algorithm on Bioconductor software to process all raw data files to obtain the expression value of each gene chip. For the miRNA microarray, qualified human plasma miRNA datasets were imported into the online tool GEO2R.

### Quality control and DE-mRNA screening

For quality control (QC), we used the Relative Log Expression (RLE) method to load the included mRNA expression datasets. RLE establishes a reference array that is generated from the median of all arrays for each probe set, and the expression value of each sample was normalized. Most of the expression values are supposed to be stable with respect to the median and should be approximately 0, accordingly.

The “Batch effect” is a type of non-biological expression variation that is found across multiple batches of microarray analysis, making it difficult to combine data for an integrated analysis.

Johnson WE et al. proposed parametric and nonparametric empirical Bayes frameworks to adjust data for batch effects that are robust to outliers in small sample sizes, making them comparable to large sample methods [10]. We used this method to remove the batch effects using the Surrogate Variable Analysis (SVA) package in R studio to make the data more suitable for comparisons.

The Linear Model for Microarray (LIMMA) package was used to pool the eligible microarray data to acquire DE-genes in stroke. In LIMMA, *P*-values were extrapolated with a modified two sample t-test, and Fisher’s method was implemented to analyze differences between two groups [11]. A corrected P-value (*P* < 0.05) and log Fold Change > 1.4 were considered to be statistically significant for DE mRNAs in stroke, and a false discovery rate (FDR) of 0.05 was used to correct for multiple testing. For visualization, DE mRNAs were plotted using the MetaDE package.

### Enrichment analysis of stroke-related DE genes

Online tools, such as Database for Annotation, Visualization and Integrated Discovery (DAVID), Kyoto Encyclopedia of Genes and Genomes (KEGG) pathways, Gene Ontology (GO) terms and Genetic Association Database (GAD) were used to predict the prospective function and further functional categories [12–14]. *P* < 0.05 was considered significant in the enrichment analysis. Given that proteins are the biomacromolecules that execute functions in our bodies, the STRING database [15] was applied for the critical assessment by visualizing the Protein-Protein Interactions (PPI) using Cytoscape [16].

### Gene set enrichment analysis

Gene Set Enrichment Analysis (GSEA) is an advanced method for determining whether an a priori defined set of genes shows statistically significant, consistent differences between two biological groups [17]. This method has advantage due to focusing on gene sets, that is, groups of genes that share a common biological function, chromosomal location, or regulation. This method can avoid the limitations of the common enrichment approach, which focuses on a handful of genes at the top of L, that is, those genes which exhibit the largest difference.

### Analysis of the miRNA expression dataset and target prediction

GEO2R (http://www.ncbi.nlm.nih.gov/geo/geo2r/) is an easy-to-use online tool for identifying differential expression in miRNA series. GEO2R automatically calculates the false discovery rate (FDR) and detects statistically significant genes (*p* < 0.05) simultaneously with FDR correction by using multiple t-test.

The target genes of miRNAs were predicted using miRNet, which is a comprehensive tool suite that enables the statistical analysis and functional interpretation of data generated from current miRNA studies.

**Fig. 1:**
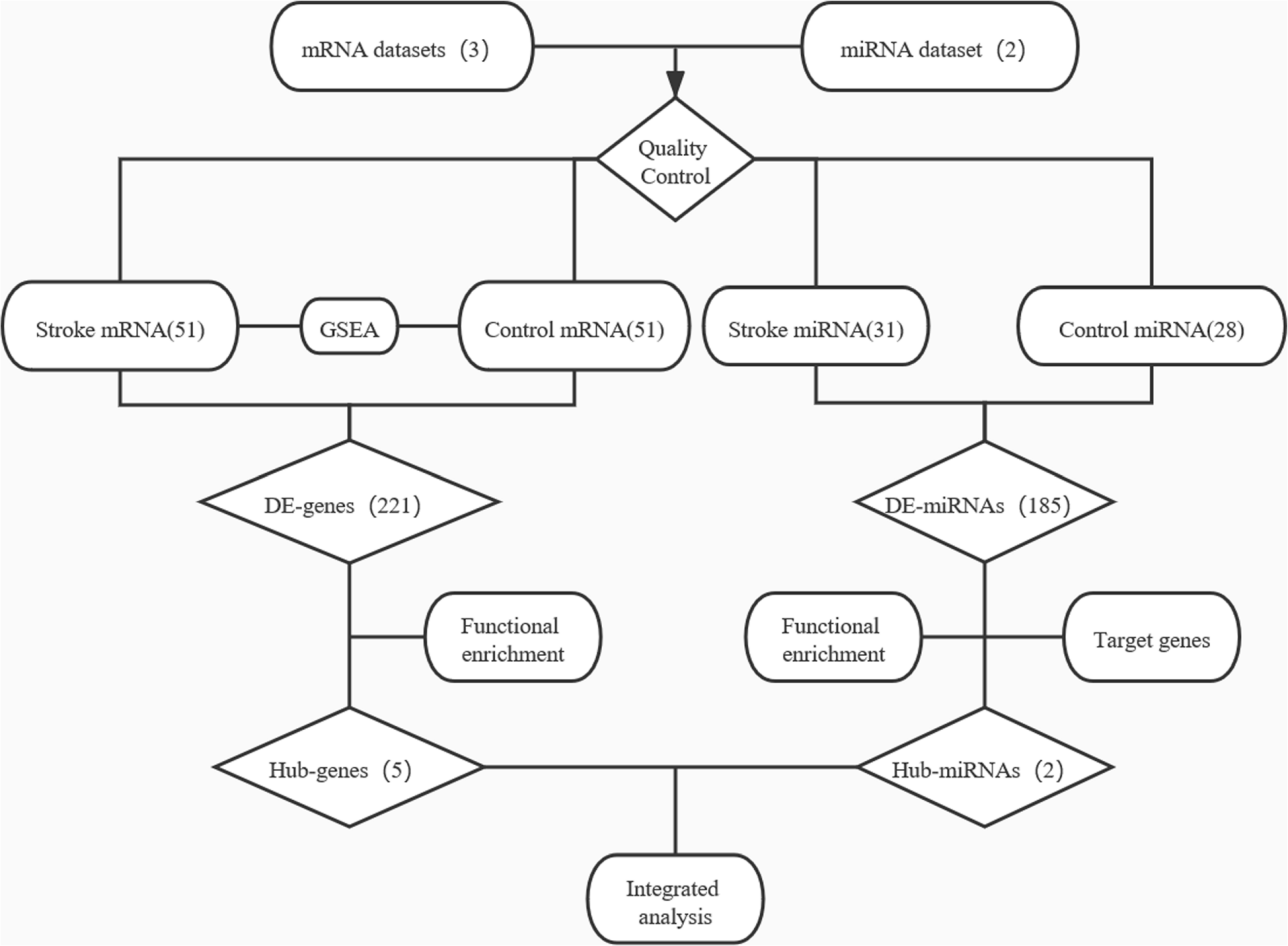
Flowchart illustrating the bioinformatic analysis process

A simplified flowchart (Fig. 1) illustrates the above-described process.

## Results

### Coanfluence analysis of ischemic stroke gene expression datasets

**Fig. 2:**
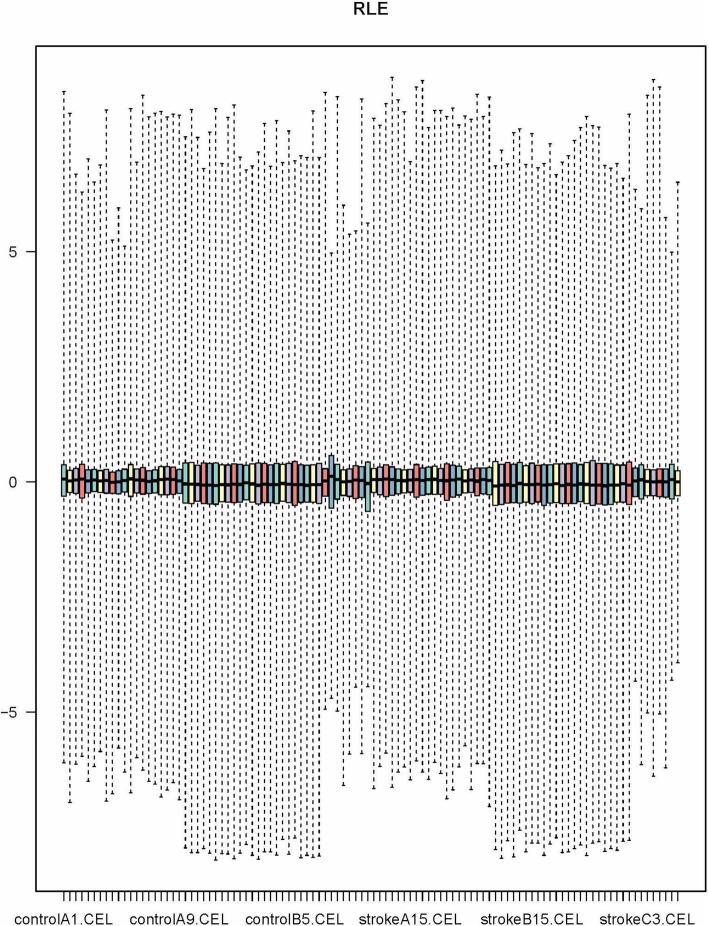
Relative Log Expression (RLE) signal graph

**Fig. 3:**
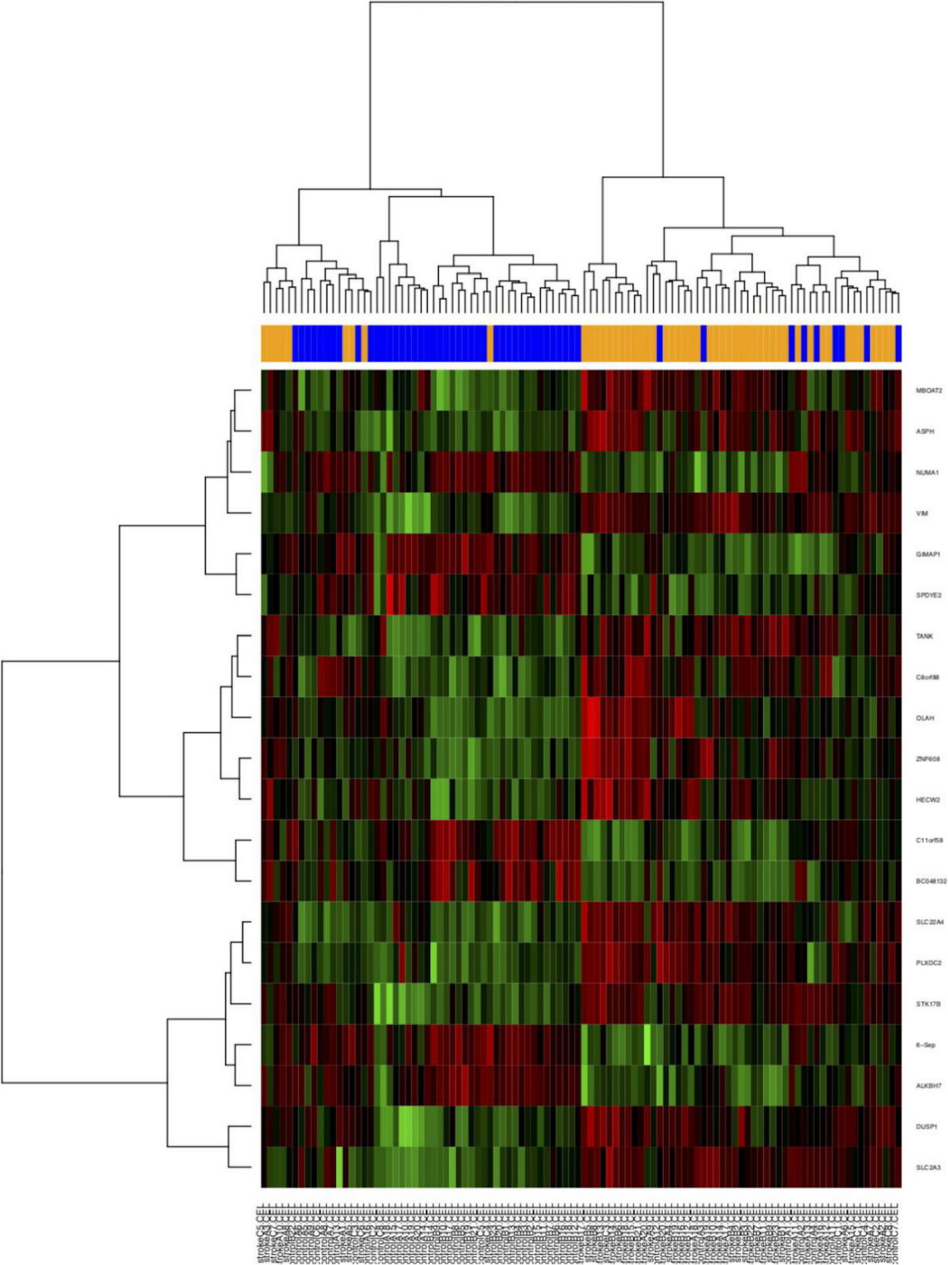
Heatmap of the top 20 differentially expressed genes (for the sake of space, only a portion of the figure is shown here)

Three primary datasets with available mRNA expression data for PBL samples in stroke patients were identified by searching the GEO database (GSE66724, GSE58294, GSE22255). The detail of the participants are provided in **additional file****2**. After quality control using RLE and the removal of the batch effect (Fig. 2), a total of 102 PBL samples (51 patients and 51 controls, Table 1) were pooled into the DE-gene analysis. Approximately 221 DE mRNAs were identified (155 upregulated mRNAs and 66 downregulated mRNAs). A heatmap of the top 20 DE mRNAs was generated by setting a specific FDR and fold change value (Fig. 3). The details of each DE mRNA are given in Supplementary table (**see** additional file 1).

**Table 1:** Baseline characteristics of datasets

Study	Country	GEO accession	Platform	Sample type	Experiment type	Case/Control		
						Number	Age (mean ± SD)	Male%
Allende M	Spain	GSE66724	GPL570	PBL^a^	mRNA	8/8	/	/
Stamova B	USA	GSE58294	GPL570	PBL	mRNA	23/23	71.7 ± 7.9/57.9 ± 3.3	52.2/52.2
Krug T	Portugal	GSE22255	GPL570	PBL	mRNA	20/20	60.2 ± 10.6/58.7 ± 11.0	50.0/50.0
Tian C	China	GSE86291	GPL18402	Plasma	microRNA	7/4	64.4 ± 16.5/59.8 ± 4.0	85.7/75.0
Jickling GC	USA	GSE55937	GPL16384	PBL	microRNA	24/24	62.4 ± 8.1/63.8 ± 8.5	50/50

^a^Peripheral blood lymphocytes

### Enrichment analysis of the DE-mRNAs

**Fig. 4:**
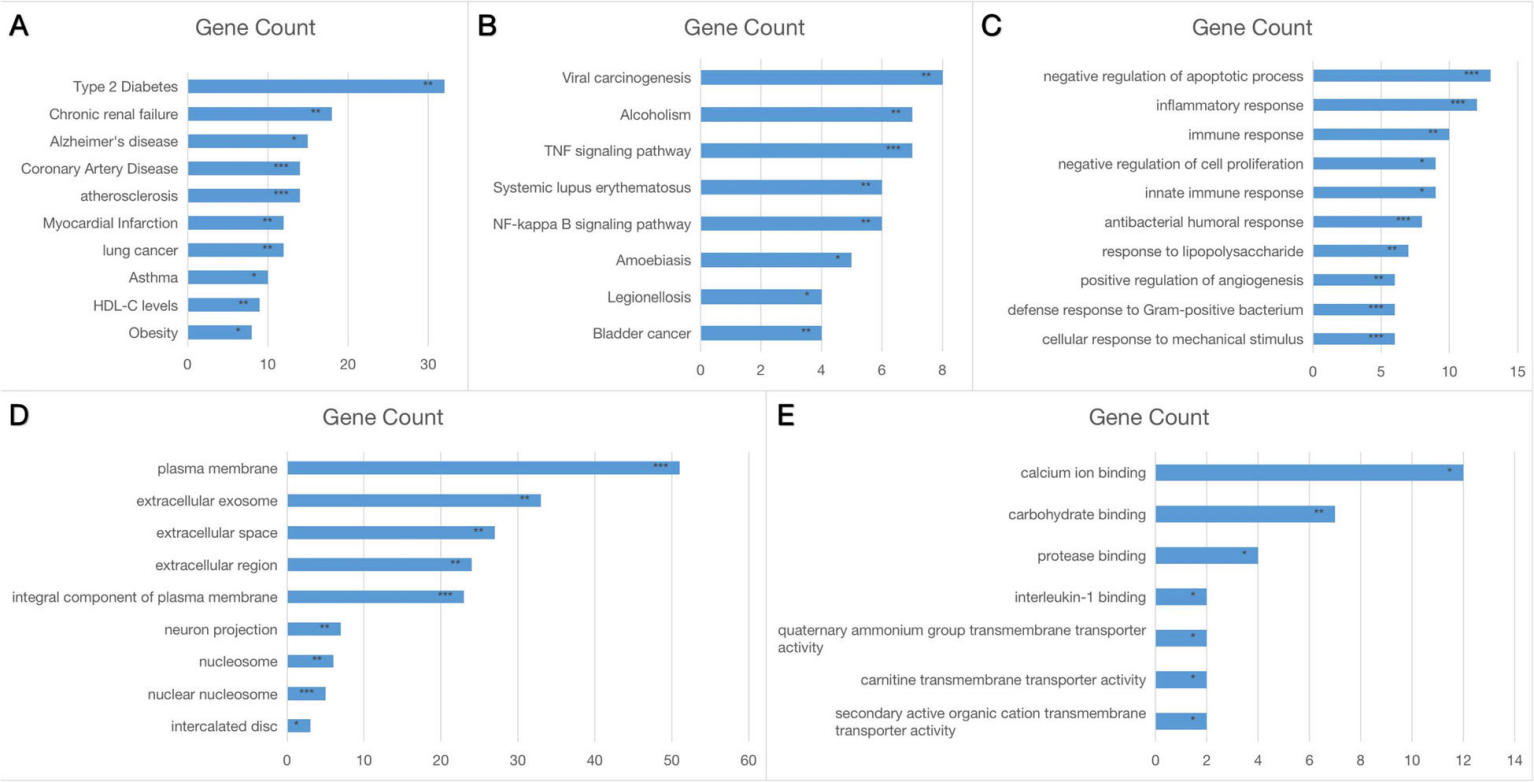
Functional enrichment analysis of meta-DE genes. (A) GAD-disease analysis. (B) KEGG pathway enrichment analysis. (C) Cellular components of GO enrichment analysis. (D) Biological processes of GO enrichment analysis. (E) Molecular functions of GO enrichment analysis

Through the enrichment analysis of Genetic Associated Disease, type 2 diabetes, chronic renal failure, Alzheimer’s disease, coronary artery disease, atherosclerosis, myocardial infarction, lung cancer, asthma, high-density lipoproteincholesterol(HDL-C) level, asthma and obesity were deemed important in stroke (Fig. 4a). In the KEGG pathway enrichment analysis, DE-mRNAs were primarily involved in viral carcinogenesis, alcoholism, the tumor necrosis factor(TNF)-signal pathway, the Nuclear Factor-KappaB(NF-kappaB) pathway and the SLE pathway. The enrichment results using the Gene Ontology(GO) database in three categories were as follows: (1) biological processes: plasma membrane, extracellular exosome and neuron projection (Fig. 4b); (2) cellular component: inflammatory response, negative regulation of cell proliferation and positive regulation of angiogenesis (Fig. 4c); (3) molecular functions: calcium ion binding, carbohydrate binding and protease binding (Fig. 4d). The above-mentioned enrichment analysis revealed that DE-genes were primarily related to common risk factors, such as type 2 diabetes, coronary artery disease and atherosclerosis, and the general event was the activation of the TNF-signaling pathway (Fig. 4e). Furthermore, we identified a pathway (the SLE pathway) that has seldom been reported to be closely associated with stroke, which was consistent with the results of the GO enrichment with immune response biological process.

**Fig. 5:**
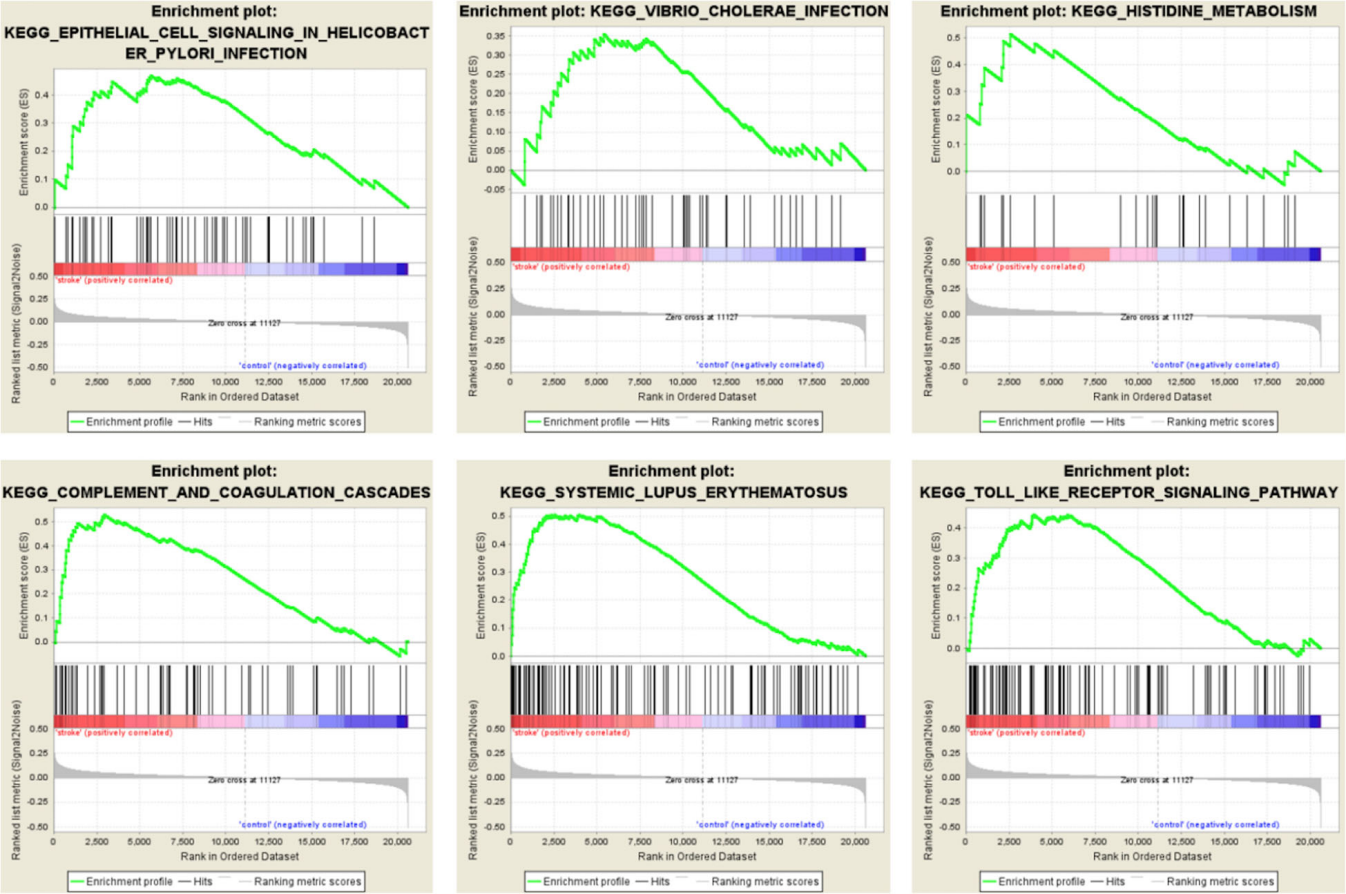
KEGG pathway enrichment by GSEA

**Fig. 6:**
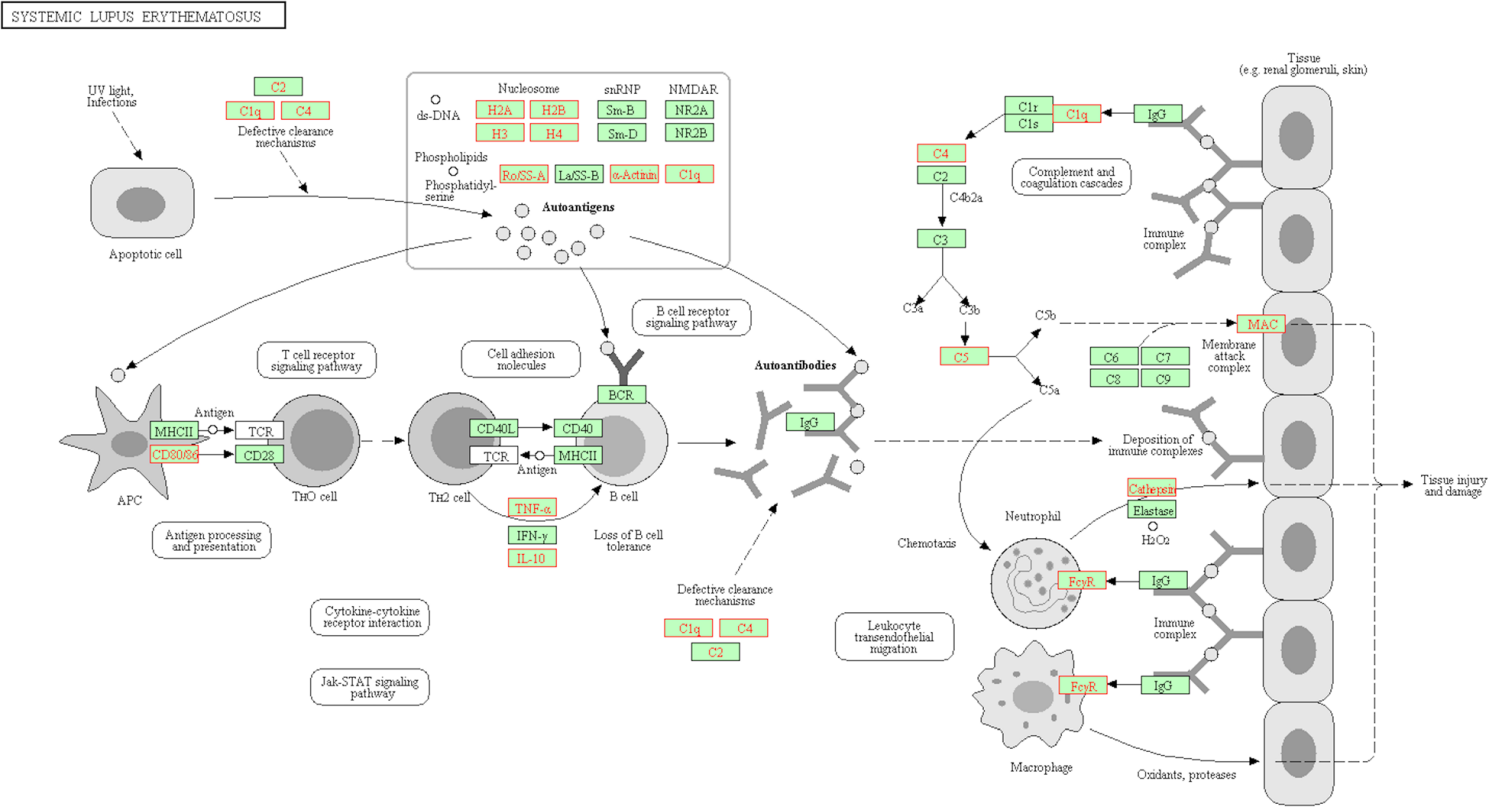
Systemic lupus erythematosus pathway in KEGG. The core enrichment genes identified in GSEA are shown in red

In the GSEA, we identified six pathways that were enriched (Fig. 5). These pathways included epithelial cell signaling in HP infection, vibrio cholerae infection, histidine metabolism, complement and coagulation cascades, systemic lupus erythematosus and the toll-like receptor signaling pathway. Notably, the SLE pathway (Fig. 6) was identified in both the DE-gene analysis and the GSEA analysis, indicating that this pathway may be strongly related to stroke.

**Fig. 7:**
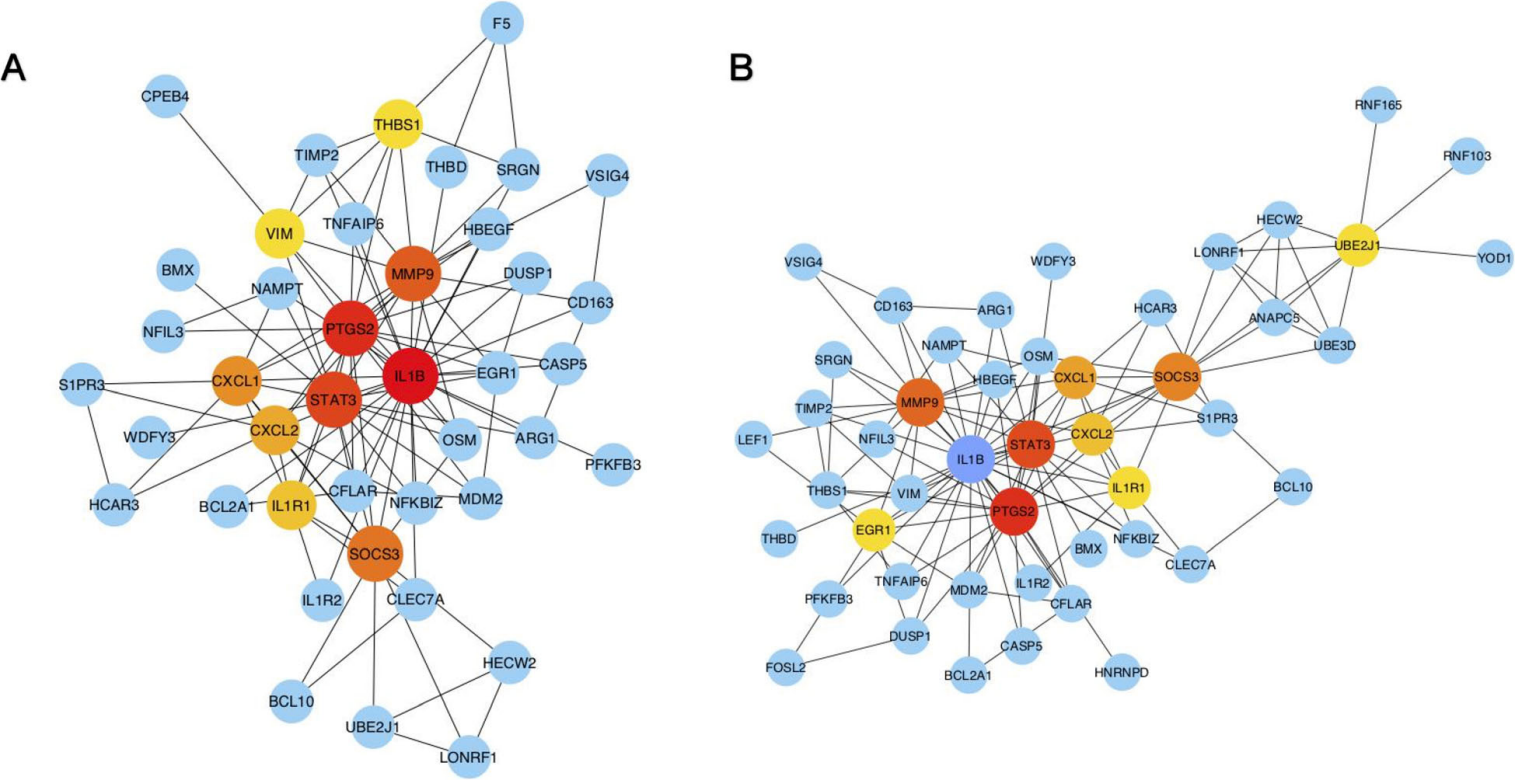
PPI networks. (A) PPI network of upregulated DE-mRNAs. (B) PPI network of all DE-mRNAs. The size and color of the map nodes are determined by the degree value; a small size with a low degree is shown in blue, and a large size with a high degree is shown in red

To further investigate the functions and interactions of the upregulated DE-mRNAs (Fig. 7) and all DE-mRNAs, we used the STRING database to construct two PPI networks, and the results were imported into Cytoscape and visualized. Interestingly, six genes (*PTGS2*, *IL1B*, *STAT3*, *MMP9*, *SOCS3* and *CXCL1*) were located in the central position of the PPI networks. The significance level is shown in Table 2. Of these core genes, five of them (excluding *STAT3*) were linked to the TNF signaling pathway.

**Table 2:** Significance levels of the six core genes

Probe ID	Gene	logFC	AveExpr	t	P.Value	adj.P.Val	B
1554997_a_at	PTGS2	−1.24882193	7.442496698	−4.80511919	5.16876E-06	0.000540758	3.864008467
205067_at	IL1B	−0.698039222	10.48159159	−3.076232156	0.002673054	0.027765348	−1.836198698
243213_at	STAT3	−0.577032001	6.221396911	−4.567697671	1.34764E-05	0.000947071	2.97764575
203936_s_at	MMP9	−0.605072277	8.96615712	−3.402170393	0.000946897	0.014217238	−0.90791425
227697_at	SOCS3	−0.688732269	9.644451144	−3.821735382	0.000224833	0.005527334	0.394351896
204470_at	CXCL1	−0.665002791	7.462440332	−3.129305647	0.002268543	0.025006572	−1.690241743

### Analysis of the stroke miRNA expression dataset

**Fig. 8:**
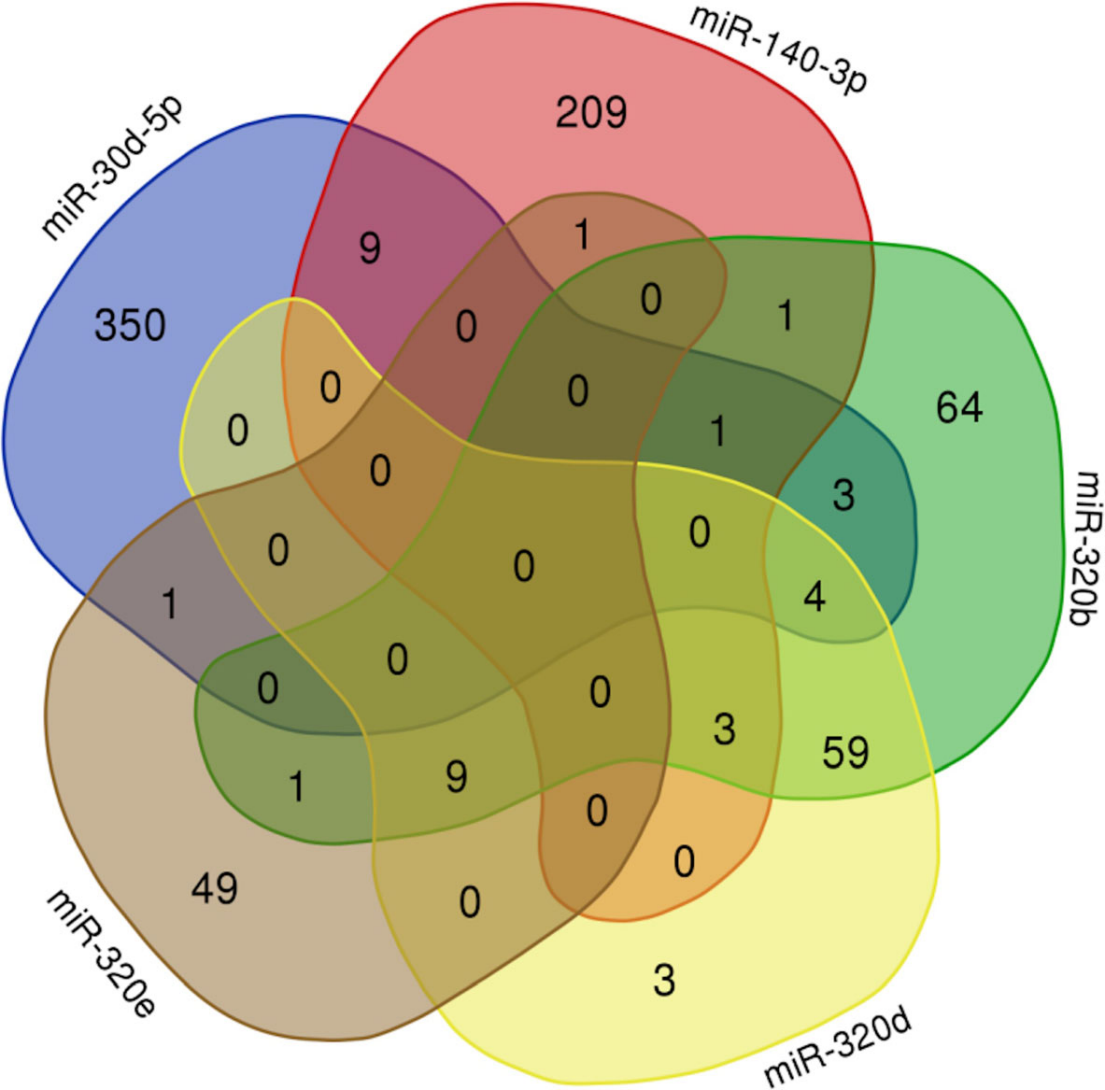
Intersection of the target genes of the top five miRNAs

**Fig. 9:**
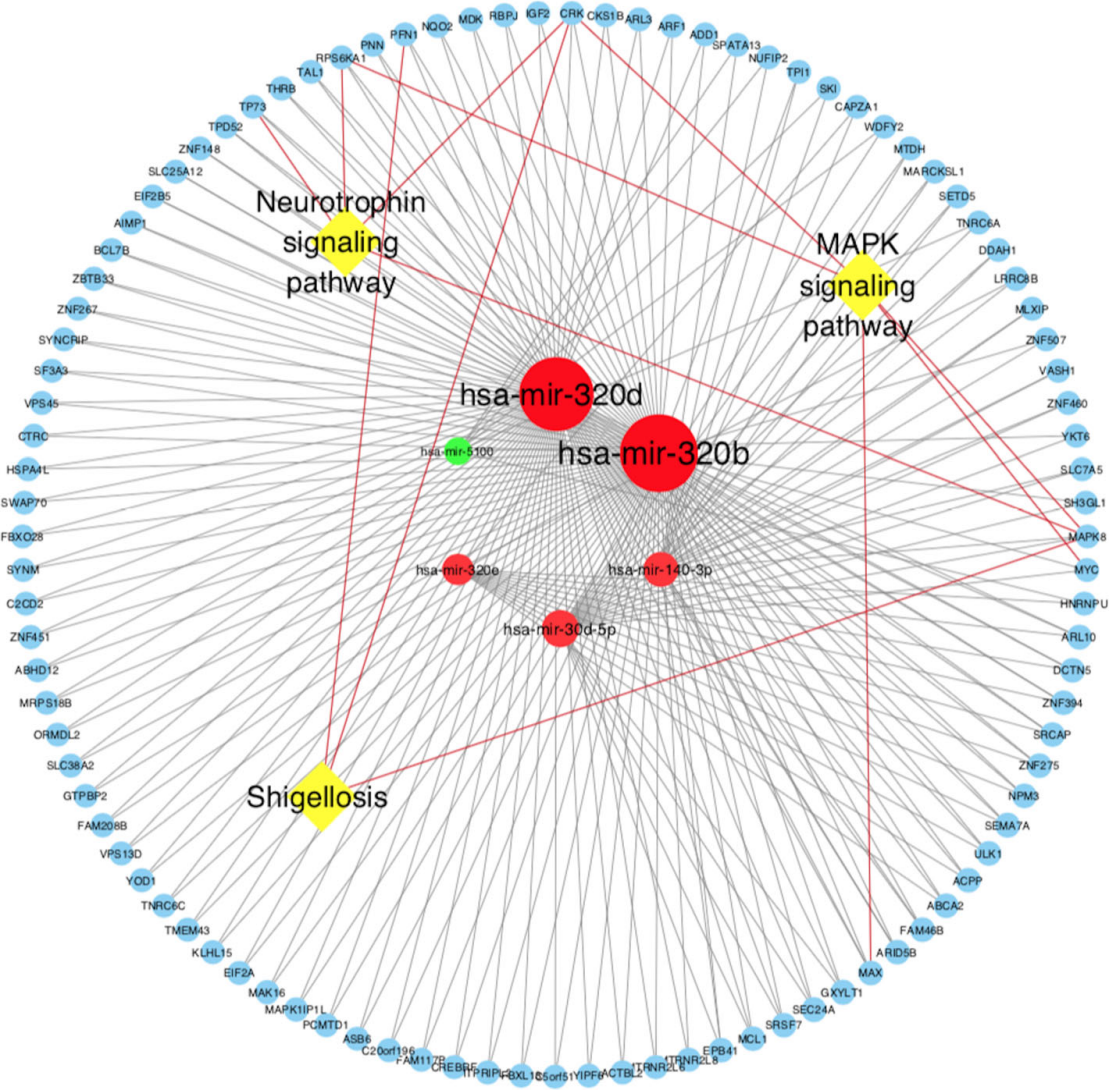
Outline of the interactions among the significant KEGG pathways, DE genes and miRNAs

Dataset GSE86291 and GSE55937 were available miRNA expression datasets containing 59 plasma samples. 31 samples were from stroke patients, and 28 were from control groups. After QC (Fig. S1), a total of 185 DE-miRNAs were identified using the online tool GEO2R(74 DE-miRNAs from GSE86291 and 111 DE-miRNAs from GSE55937). Since they were generated from different platform, we were not supposed to compare the data directly. But they actually shared one miRNA in common, that is has-miR-3135b. Among GSE86291, the top six most significant DE-miRNAs were hsa-miR-140-3p, hsa-miR-320b, hsa-miR-320d, hsa-miR-320e, hsa-miR-5100 and hsa-miR-30d-5p (Table 3). An experimentally supported miRNA database (miRBase) was used to predict the target genes of the identified miRNAs base on experimental verification and various prediction algorithms. Target genes that were regulated by two or more DE-miRNAs were included to form the miRNA-target gene-pathway network (Fig. 8 and Fig. 9). Through this network, three pathways, including the Neurotrophin signaling pathway, Mitogen-activated protein kinase(MAPK) signaling pathway and Shigenllosis infection were presented. Notably, miR-320b and miR-320d had the most common target genes, which made these miRNAs the center of the regulatory network.

**Table 3:** DE-miRNAs

miRNA ID	P.Value	t	B	logFC	Regulation
hsa-miR-140-3p	0.00194	4.12368	−2.43	4.81174	up
hsa-miR-320b	0.0024	3.9932	−2.51	4.83185	up
hsa-miR-320d	0.00309	3.83947	−2.61	4.96841	up
hsa-miR-320e	0.00438	3.63176	−2.75	4.64221	up
hsa-miR-5100	0.00654	−3.39579	−2.91	−1.86679	down
hsa-miR-30d-5p	0.00683	3.37023	−2.93	5.55511	up
hsa-miR-320a	0.00717	3.34187	−2.95	4.38005	up
hsa-miR-4454	0.00893	−3.21442	−3.05	−2.17604	down
hsa-miR-3195	0.00959	3.17256	−3.08	4.14651	up
hsa-miR-6090	0.0112	3.08296	−3.15	1.03161	up
hsa-miR-2392	0.01162	3.06168	−3.16	3.29409	up
hsa-miR-642b-3p	0.01246	3.02149	−3.19	4.24362	up
hsa-miR-106b-5p	0.01257	3.01629	−3.2	4.97951	up
hsa-miR-4687-3p	0.01307	2.99373	−3.21	0.33535	up
hsa-miR-149-3p	0.01621	−2.86985	−3.31	−0.34278	down
hsa-miR-345-5p	0.01659	−2.85646	−3.32	−0.34535	down
hsa-miR-3135b	0.01675	2.85072	−3.33	3.88918	up
hsa-miR-622	0.01766	−2.82032	−3.35	−0.35244	down
hsa-miR-513c-5p	0.01849	−2.79399	−3.37	−0.35776	down
hsa-miR-583	0.01901	−2.7779	−3.38	−0.36108	down

### Comprehensive analysis of DE genes and miRNAs

Overall, we identified potentially useful biomarkers, six mRNAs and two miRNAs, as well as several novel pathways (the SLE pathway and atypical infection pathways) as a matter of priority.

## Discussion

Cross-country studies of ischemic stroke gene expression datasets were standardized and integrated in our study using a precise method for further integrated analysis. The purpose of our work was to reduce the bias of sample studies and to screen for significant susceptibility genes that may be used to predict the potential for stroke. We used the MetaDE package in R language to merge and filter gene expressions [18]. A total of 155 upregulated DE mRNAs in PBL samples of stroke and 185 DE miRNAs were identified. Of these, we identified six genes (*PTGS2*, *IL1B*, *STAT3*, *MMP9*, *SOCS3* and *CXCL1*) and two miRNAs (miR-320b and miR-320d) as worth exploring due to their core position in the network and the functional enrichment.

Some of the above-mentioned DE-miRNAs were confirmed to be involved in the pathophysiological process of stroke, including neurogenesis (miR-30-5p [19]), neuroprotection (miR-223 [20], miR-424 [21] and miR-106-5p [22]) and angiogenesis (miR-130a [23]). To verify the identified DE-miRNAs in depth, we predicted the target genes and enrichment pathways of the top five DE-miRNAs. ultimately, we speculated that miR-320b and miR-320d were more likely to compensate the pathophysiological process of stroke through the neurotrophin signaling pathway.

The enrichment analysis from the GAD database revealed that traditional risk factors play an important role in the onset of stroke, including type 2 diabetes, heart disease, atherosclerosis, high HDL-C levels and Obesity. In addition, the KEGG enrichment and GSEA revealed that DE genes were primarily involved in the TNF and SLE pathways as well as in atypical microbial infection (virus, amoebiasis, legionellosis, vibrio cholerae); these findings were consistent with the inflammatory and immune dysfunction categories in the results of the GO enrichment with biological process. Inflammation and immunity are key elements of the pathobiology of stroke. The immune system gets involved in the cerebral ischemic damage, and the damaged brain in turn suppresses immunity, thereby increasing the incidence of infections and poor outcomes. Inflammation signaling participates in the overall process of the ischemic cascade, from the initial damaging events triggered by arterial occlusion to the late regenerative process underlying post-ischemic tissue repair [24]. Combining the results of the two PPI networks, five core genes (*PTGS2*, *IL1B*, *SOCS3*, *MMP9* and *CXCL1*) were linked to inflammation and immunity.

In the early stage of stroke, damaged neurons and endothelial cells produce COX-2 (encoded by *PGTS2*), which is an important source of prostaglandin. Prostaglandin is a vital inflammatory mediator that launches inflammation and alters the permeability of the blood brain barriers [25]. Subsequently, the microglia in the central nervous system and macrophages in the perivascular space release cytokines, such as TNF and IL-1β (encoded by *IL-1B*, providing further signals to guide leukocyte migration across the vascular wall) [26]. The chemokine (C-X-C motif) ligand 1 (*CXCL1*) is a small cytokine belonging to the CXC chemokine family and is expressed in epithelial cells, macrophages and neutrophils, helping to recruit leukocytes to the damaged endothelial cells [27]. When leukocytes migrate from the open blood brain barriers to the vessel extracellular matrix, Matrix metallopeptidase 9 (*MMP-9*) is activated to break down the extracellular matrix and remodel it to facilitate the migration of leukocyte to the focus. In the period following stroke, the inflammation responses that clear the dead cells also cause tissue damage and the activation of innate and adaptive immunity [28]. Moreover, recent research shows that vascular endothelial growth factor (VEGF) is crucial for post-ischemic angiogenesis and is produced by activated astrocytes; fully functional VEGF may require MMPs, suggesting a link between inflammatory cells and angiogenesis [29]. As for signal transducer and activator of transcription 3 (*STAT3*), this neuroprotective factor is essential for the differentiation of Th17 cells and for maintaining the ability to generate antibodies of adaptive immunity.

However, high expression of *STAT3* in microglia was shown to play a critical role in mediating Hcy-induced microglia activation and neuroinflammation in a rat middle cerebral artery occlusion (MCAO) model [30]. Therefore, the role of *SATA3* in stroke is still controversial. In addition, suppressor of cytokine signaling 3 (*SOCS3*) has been identified to have an emerging role linking central insulin resistance and Alzheimer’s disease, but the relationship between *SOCS3* and stroke has not been studied sufficiency [31].

The KEGG and GAD enrichment analyses for DE-genes revealed that the DE-genes related to the following three types of diseases: (1) type 2 diabetes, atherosclerosis and coronary artery disease, which all represent vascular endothelial injury caused by metabolic disorders and fatty acid accumulation; all are considered high risk factors for stroke; (2) SLE and asthma, which both involve excessive inflammation and immune response; and (3) microbial infections (helicobacter pylori, virus, amoebiasis, legionellosis, vibrio cholerae), which are all a direct consequence of immunosuppression in late post-ischemic stroke. The prevailing conclusion is that stroke is a polygenic condition made our integrated analysis more effective and valid.

Here, we proposed that the SLE pathway may be a rare stroke-related pathway. This pathway has been reportedly linked to cerebral lupus, especially epilepsy and acute psychotic disorder [32]. It has been reported that stroke represents one of the most severe complication, with an occurrence rate between 3 to 20%, particularly in the first 5 years of diseases [33]. The mechanisms underlying SLE and stroke involve the expression of aPL (a common SLE antibody) on endothelial surfaces, which leads to the release of pro-inflammatory cytokines and the upregulation of adhesion molecules [34]. However, it is not clear how these antibodies trigger thrombosis. In our study, we outlined the upregulated proteins of the SLE pathway. In the generation stage of auto-antibodies, the overexpression of CD80/86 in antigen-presenting cells accelerates the transduction from Th0 cells to Th2 cells. Then, Th2 cells assist the B cells in producing more antibodies. In the effective stage, on the one hand, C4/C1q/C5 in the complement pathway is activated by the antigen-antibody complex to form the MAC (membrane attack complex), leading to vascular endothelial injury. On the other hand, recruited neutrophil granulocytes and macrophages secrete cathepsin, leading to tissue damage in the brain [35].

Another issue we would like to explore is the atypical infections found in our study. Infection occurrence is an critical trigger that precedes up to one third of ischemic strokes, and infections that present subsequent to ischemic stroke also complicate one third of the cases and bring about worse outcomes [36]. One of the largest studies, which included 19,063 first-time stroke patients, indicated that the risk of stroke was highest during the first 3 days after the diagnosis of respiratory tract infection (IR = 3.19 95%CI 2.81–3.62) or urinary tract infection (IR = 2.72 95%CI 2.32–3.20). However, in the following PASS (preventive antibiotics in stroke study), preventive ceftriaxone did not improve functional outcomes in patients with acute stroke [37]. In our study, the most significant infections were *Helicobacter pylori(HP)*, virus and certain atypical microorganisms, including amoebiasis, legionellosis and vibrio cholerase, which were not covered by ceftriaxone. Several retrospective analyses have shown that HP infection is associated with stroke, but their conclusions were contradictory [38]. In addition, several viral infections (cytomegalovirus, Herpes simplex virus 1, varicella zoster virus, hepatitis C virus and human immunodeficiency virus) have been implicated in increasing the risk of ischemic stroke. However, the more atypical infections found in our study were not covered by ceftriaxone, which may account for the negative results in PASS. Notably, there is a lack of research on the relationships between these infections and stroke. With the rise in research on the gut-brain axis, it has been shown that stroke promotes the translocation and dissemination of selective bacterial strains that originate from the host intestinal microbiota [39]. Moreover, the velocity of stroke induces intestinal barrier dysfunction and permeability more rapidly than the dissemination of orally ingested bacteria to peripheral tissues. These studies raised our awareness that we should pay more attention to the relationship between stroke and inapparent infections of the digestive system [40].

Our study has several limitations. The first was the sample types of mRNA datasets. In the original protocol, we aimed to obtain data from blood, cerebrospinal fluid and brain samples in order to restore the differences in gene expression from the periphery and center to pathology. However, due to the limitation of datasets and the inaccessibility of the raw data, the sample type was restricted to blood sample data only. The second was the lack of compare of two miRNA datasets from different platform. We just listed the results of the individual analysis together but could not overcome the differences between the platforms to do the fusion analysis. Jung KC and Daniel R [41, 42] developed a random effects model shown to be appropriate for gene express datasets, independent of the method and technology used(ie, spotted cDNA versus oligonucleotide). What’s more, By using this method, dataset from different experiment type and platform could cross-verify each other, and that will greatly increased the credibility of microarray analysis. And we were looking forward to see more breakthrough in miRNA analysis in the future. The last shortcoming is that the causal relationship between the novel biomarkers and stoke can only be predicted by theoretical analysis rather than through prospective study. Therefore, we will keep monitoring the progress in stroke research. Further investigations are warranted to confirm whether our novel biomarkers are potential prognostic predictors or therapeutic targets in stroke.

## Conclusion

Our integrated analysis of stroke genomics provides abundant resources for further explorations of the role of target genes and miRNA in ischemic stroke. Six significantly upregulated genes (*PTGS2*, *IL1B*, *STAT3*, *MMP9*, *SOCS3* and *CXCL1*) and two significantly upregulated miRNAs (miR-320b and miR-320d) were identified as potentially useful clinical diagnostic markers. Systemic Lupus Erythematosus pathways and atypical pulmonary and digestive infections may participate the pathogenesis of stroke; therefore, these topics warrant further study.

## Supplementary information

**Additional file 1: Table S1.** The DE-miRNA of two groups of miRNA dataset.

**Additional file 2: Fig. S1.** The boxplot of miRNA sample.

## Data Availability

The datasets generated and/or analyzed during the current study are available from the GEO database and can be download through the following link. https://www.ncbi.nlm.nih.gov/geo/query/acc.cgi?acc=GSE66724 https://www.ncbi.nlm.nih.gov/geo/query/acc.cgi?acc=GSE22255 https://www.ncbi.nlm.nih.gov/geo/query/acc.cgi?acc=GSE86291 https://www.ncbi.nlm.nih.gov/geo/query/acc.cgi?acc=GSE58294 https://www.ncbi.nlm.nih.gov/geo/query/acc.cgi?acc=GSE55937

## Abbreviations

CXCL1: The chemokine (C-X-C motif) ligand 1
DE: Differential expression
DAVID: Database for annotation, visualization and integrated discovery
GEO: Gene expression omnibus
GO: Gene ontology
GSEA: Gene set enrichment analysis
GAD: Genetic association database
HDL-C: High-density lipoproteincholesterol
IS: Ischemic stroke
IL1B: Interleukin-1 beta
KEGG: Kyoto encyclopedia of genes and genomes
LIMMA: Linear model for microarray
mRNA: Messenger RNA
miRNA: microRNA
MMP9: Matrix metallopeptidase 9
MAC: Membrance attack complex
PTGS2: Prostaglandin-endoperoxide synthase 2
PPI: Protein-protein interaction
QC: Quality control
PASS: Preventive antibiotics in stroke study
SLE: System lupus erythematosus
RLE: Relative log expression
SOCS3: Suppressor of cytokine signaling 3
