# Supplementary figures and images for: Identification of novel biomarkers in ischemic stroke: a genome-wide integrated analysis
Source: BMC Med Genet. 2020 Mar 30;21:66. doi: 10.1186/s12881-020-00994-3 (PMC7106706; doi:10.1186/s12881-020-00994-3)

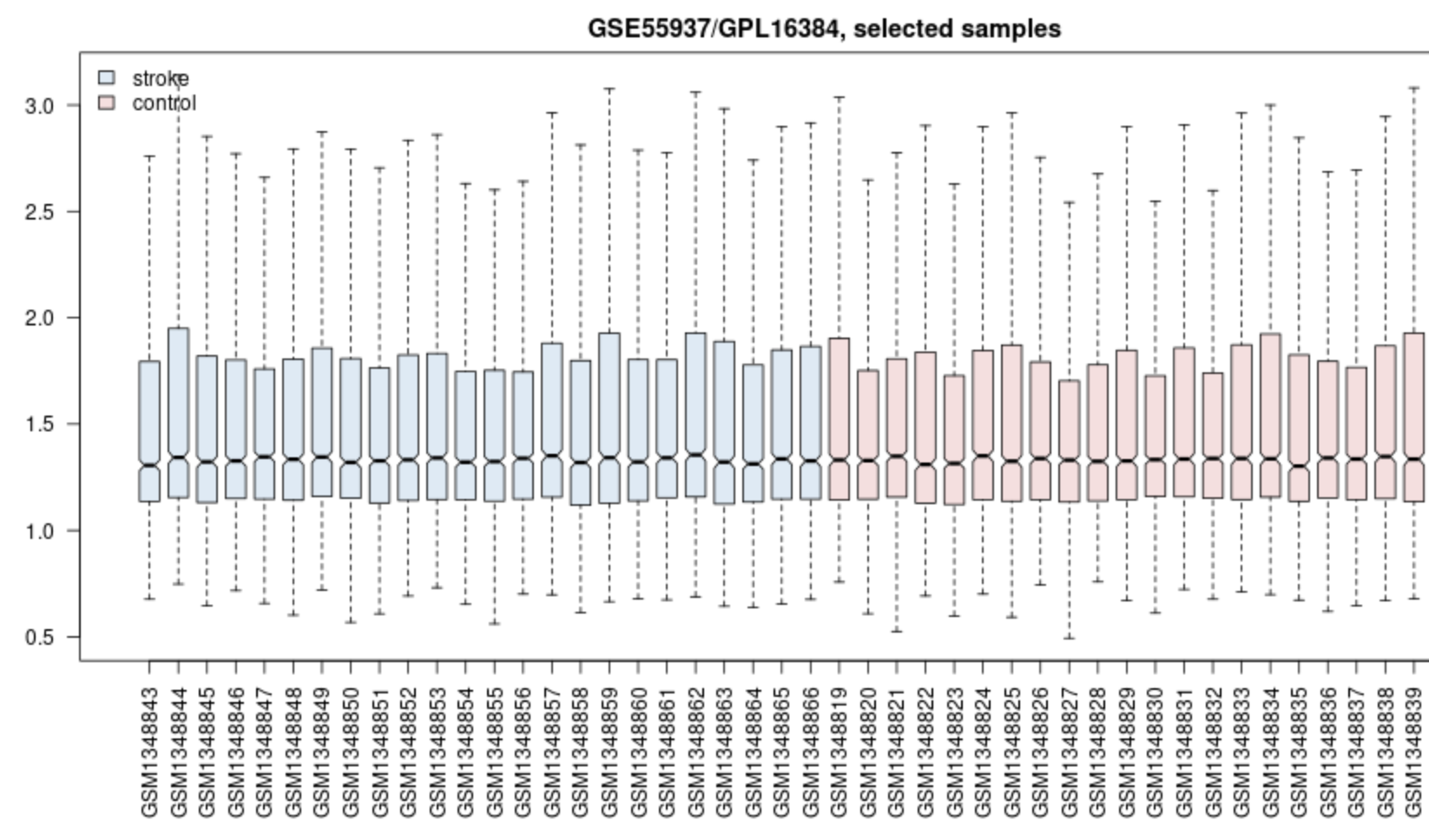

Supplement: Supplementary file 2 — Additional file 2: Fig. S1. The boxplot of miRNA sample. [file 12881_2020_994_MOESM2_ESM.png]
